# Association between IL-17, IL-23 with neurocognitive scales in patients with Alzheimer’s disease

**DOI:** 10.1192/j.eurpsy.2021.1903

**Published:** 2021-08-13

**Authors:** I. Mudrenko, O. Chyniak

**Affiliations:** Department Of Neurosurgery And Neurology, Sumy State University, Sumy, Ukraine

**Keywords:** Alzheimer’s disease, inflammation, interleukin, neurocognitive scales.

## Abstract

**Introduction:**

Alzheimer’s disease (AD) is a degenerative brain disease and the most common cause of dementia. Evidence suggests that various cytokines, including interleukins (IL) IL-6, IL-10, IL-12 are actively involved in the pathogenesis of AD. The role of IL-17 and IL-23 is less clear.

**Objectives:**

To investigate the correlations between IL-17, IL-23, and neurocognitive scales in patients with Alzheimer’s disease.

**Methods:**

The study included 45 patients: 15 patients with Alzheimer’s disease and 30 patients without cognitive deficit (control group). Clinical and psychometrical methods were used: Mini Mental State Examination (MMSE) scale; Montreal Cognitive Assessment (MoCA), Frontal Assessment Battery (FAB), Alzheimer Disease Assessment Scale-cognitive (ADAS – cog). Serum levels of cytokines of IL-17 and IL-23 were analyzed by sandwich ELISA on “Chem Well 2900” immunoanalyzer (Awareness Technology, USA).

**Results:**

A significantly positive correlation was observed between IL-17 and IL-23 for all AD patients (r =0.723, p=0.002). A significant inverse correlation was observed between serum concentration of IL-17 and MoCA score (r=˗1.0, р≤.0001) and IL-23 and MMSE score (r=˗0.553, р=0.032) in all AD patients. However, no other significant correlations were found between IL-17 and the scores MMSE, FAB, ADAS – cog and between IL-23 and the scores MoCA, FAB and ADAS – cog.

**Conclusions:**

Proinflammatory cytokines (such as IL-17 and IL-23) have been associated with cognitive impairment. However, the complicated relationships of the two cytokines with the pathogenesis of AD need to be further investigated in the future.

**Disclosure:**

No significant relationships.

